# Supplementary material for: Occurrence and Ecological Risks of Neonicotinoids in Wheat, Corn and Rice Field Soils in China
Source: Molecules. 2025 Apr 17;30(8):1803. doi: 10.3390/molecules30081803 (PMC12029873; doi:10.3390/molecules30081803)
Supplement: Supplementary file 1 [file molecules-30-01803-s001.zip › molecules-3560915-supplementary.pdf]

Occurrence and Ecological Risks of Neonicotinoids in Wheat, Corn and Rice Field Soils in China

Junxue Wu †, Pingzhong Yu †, Ziyu Zou, Ercheng Zhao *, Junjie Jing, Jinwei Zhang, Yan Tao, Lirui Ren, Min He, Li Chen and Ping Han *

Beijing Key Laboratory of Environment Friendly Management on Fruit Diseases and Pests in North China, Key Laboratory of Environment Friendly Management on Fruit and Vegetable Pests in North China (Co-Construction by Ministry and Province), Ministry of Agriculture and Rural Affairs, Institute of Plant Protection, Beijing Academy of Agriculture and Forestry Sciences, Beijing 100097, China; wujunxue@baafs.net.cn (J.W.); yupingzhong@baafs.net.cn (P.Y.); ziyu2965@163.com (Z.Z.); jingjunjie@baafs.net.cn (J.J.); zhangjinwei@baafs.net.cn (J.Z.); taoyan@baafs.net.cn (Y.T.); renlirui@baafs.net.cn (L.R.); hemin@baafs.net.cn (M.H.); chenli@baafs.net.cn (L.C.)

* Correspondence: eczhao@126.com (E.Z.); hanping@baafs.net.cn (P.H.)

† These authors contributed equally to this work.

Tables

Table S1. Properties (solubility, log K_{ow}, log K_{oc} and log K_d) and environmental persistence of the NEOs

Table S2. Linear range of matrix matched calibration curves, LOD, LOQ, spiked average recoveries and relative standard deviations (RSDs) of the NEOs

Table S1. Properties (solubility, log K_{ow}, log K_{oc} and log K_d) and environmental persistence of the NEOs

Neonic	Vapor Pressure (mPa) @ 20 °C	Water Solubility (mg/L) @ 20 °C	Log K_{ow} [a]	Log K_{oc} [a]	Log K_d [a]	Soil Persistence (DT50 in days)	Water-sediment (DT50 in days)	Water Photolysis (DT50 in days)	Water Hydrolysis (DT50 in days)
IMI	4.0E-7	610	0.57	2.19-2.90	1.20	191 (174-191)	30-129 (stable)	< 1; 0.2 (fast)	> 365 (stable)
THX	6.6E-6	4100	-0.13	1.75	0.37	50 (7-72)	31-40 (stable)	2.7-39.5 (moderately fast)	11.5 (stable)
CLO	1.3E-7	340	0.91	2.08	1.20	545 (13-1386)	40-56 (stable)	< 1; 0.1 (fast)	14.4 (stable)
DIN	1.7E-3	39830	-	26	-	82	-	-	
ACE	1.0E-3	2950	0.80	2.3	1.32	3 (2-20)	4.7 (moderately fast)	34 (stable)	420 (stable)
THIA	3.0E-7	184	1.26	3.67	1.45	15.5 (9-27)	8-28 (stable)	10-63 (stable)	n/a (stable)
IMTH	-	-	-	-	-	3.1	-	-	-
NIT	1.1E-3	590000	-	60	-	8	-	-	(n/a)(stable)
CYC	-	-	-	-	-	-	-		
PCD	-	-	-	-	-	1.7(1.7-3.15)	-	-	

Data source: Pesticide Products Database (PPDB), available at: PPDB - Pesticides Properties DataBase (herts.ac.uk)

n/a: no data available

Table S2. Linear range of matrix matched calibration curves, LOD, LOQ, spiked average recoveries and relative standard deviations (RSDs) of the NEOs

Pesticides	Range of curve (μg/kg)	calibration curve	*r*	LOD (μg/kg)	LOQ (μg/kg)	Range of spiked recovery/(μg/kg)	Recovery (%)	RSDs (%)
IMI	0.1-100	y = 9637.4x + 25896	1	0.003	0.01	1, 10, 100	99.30-113.13	3.15-8.16
CLO	0.1-100	y = 6872.8x – 1385.2	0.999	0.02	0.08	1, 10, 100	95.43-107.47	3.35-6.25
TMX	0.1-100	y = 4425.9x – 201.64	0.999	0.003	0.01	1, 10, 100	99.00-113.50	5.45-12.21
DIN	2-100	y = 2178.5x + 611.92	0.999	0.5	1.7	5, 20, 100	71.43-76.40	1.14-2.36
ACE	0.1-100	y = 23678x -7909.5	0.999	0.03	0.1	1, 10, 100	95.27-101.83	2.79-4.68
THIA	1-100	y = 27148x - 10936	0.999	0.06	0.2	1, 10, 100	100.93-124.20	3.32-7.42
IMTH	1-100	y = 11049x -2490.6	0.999	0.2	0.7	1, 10, 100	100.4-119.80	3.13-11.13
NIT	1-100	y = 56971x – 8360.7	1	0.3	1.0	1, 10, 100	61.80-80.37	0.48-3.99
CYC	1-100	y = 50938x +6384.9	0.999	0.3	1.0	1, 10, 100	88.63-99.73	5.20-5.53
IPP	1-100	y = 93649x - 11079	0.999	0.1	0.3	1, 10, 100	98.77-106.00	0.98-4.59
